# From hand to mouth: monkeys require greater effort in motor preparation for voluntary control of vocalization than for manual actions

**DOI:** 10.1098/rsos.180879

**Published:** 2018-11-28

**Authors:** Hiroki Koda, Takumi Kunieda, Takeshi Nishimura

**Affiliations:** Primate Research Institute, Kyoto University, Kyoto, Japan

**Keywords:** speech evolution, primate vocalization, operant conditioning, voluntary control

## Abstract

Voluntary control of vocal production is an essential component of the language faculty, which is thought to distinguish humans from other primates. Recent experiments have begun to reveal the capability of non-human primates to perform vocal control; however, the mechanisms underlying this ability remain unclear. Here, we revealed that Japanese macaque monkeys can learn to vocalize voluntarily through a different mechanism than that used for manual actions. The monkeys rapidly learned to touch a computer monitor when a visual stimulus was presented and showed a capacity for flexible adaptation, such that they reacted when the visual stimulus was shown at an unexpected time. By contrast, successful vocal training required additional time, and the monkeys exhibited difficulty with vocal execution when the visual stimulus appeared earlier than expected; this occurred regardless of extensive training. Thus, motor preparation before execution of an action may be a key factor in distinguishing vocalization from manual actions in monkeys; they do not exhibit a similar ability to perform motor preparation in the vocal domains. By performing direct comparisons, this study provides novel evidence regarding differences in motor control abilities between vocal and manual actions. Our findings support the suggestion that the functional expansion from hand to mouth might be a critical evolutionary event for the acquisition of voluntary control of vocalizations.

## 1. Introduction

Human speech is a key component of the faculty of language [1–5] and involves the use of multiple integrated sets of capabilities, such as facial actions, respiratory control and the volitional control of vocal productions [2,6]. Importantly, the volitional control of vocal productions is essential for the expression of critical acoustic characteristics, such as pitch or resonance frequencies; moreover, such control is needed to intentionally regulate the timing of onset/offset during conversational speech. This control contributed to the evolutionary shift from visual to auditory-dependent communication in humans [7–9]. Volitional control of motor actions, including both vocalizations and manual actions (e.g. hand grasping and finger pinching), is coordinated by the motor cortex network [10,11]. By contrast, voluntary control of vocalizations in non-human primates is extremely limited, as they lack the motor circuitry necessary for volitional vocal control; this is typically manifested as a lack of volitional control of the larynx [12–21]. These laryngeal motor limitations suggest difficulty in controlling vocal pitch, which is primarily determined by muscle tensions among the vocal folds. This has led some researchers to support a gestural origin for language evolution, which contrasts with the hypothesis of vocal origin [22].

The dichotomy between humans and monkeys is consistent with human vocal ontogeny. Models of the developmental pathway to spoken language comprise two primary systems: crying and coo-babbling systems [23,24]. In the period shortly after birth, infant vocalizations exclusively comprise crying, which is tightly coupled with emotional state, including discomfort, distress and pain [25]. The infant begins to produce acoustically resonant vocalizations, known as cooing or babbling; these are regarded as speech sounds that ultimately lead to the capability for language production with the maturation of voluntary vocal control through infant–mother interactions [26]; notably, these later vocalizations are decoupled from emotional states. Infant crying shares the same neural motor system as that of vocalization in non-human primates [12,27]. Therefore, this duality in vocal development (i.e. crying vs. cooing) has long been believed to be a characteristic unique to humans. Consequently, monkey vocalizations have been regarded as homologous to uncontrollable emotional sounds, such as infant cries, which are independent of controlled speech.

However, this notion of system dichotomy is insufficient to explain the recent progress of vocal controllability in non-human primates. A direct approach to test vocal controllability is exemplified by the attempted operant conditioning of vocalization in monkeys. Regardless of the limited neural circuitry available for motor control of vocalizations, most studies showed that monkeys could be trained to vocalize in response to the cues [28–35]. In recent studies, non-human primates have shown voluntary control of their vocalizations [4,36–43]. Apart from difficulty in pitch control, unvoiced sounds, such as whispers, could be controlled in non-human primates, revealed by evidence of whisper production in captive orangutans [44–46]. This clearly suggests that the shape of the vocal tract during respiration can be controlled, in a manner distinct from laryngeal control. Vocalizations by common ancestral primates did not follow a linear evolutionary path to reach modern human speech; instead, they involved the integration of multiple domains of motor control. This stepwise evolutionary process has remained unclear in primate lineages.

Here, we focused on motor control of vocal timing, an important component for speech. In the ‘direct connections’ hypothesis, which suggests that direct connections from the motor cortex onto terminal motor neurons provide enhanced voluntary control over action, learning control of motor timing would also depend on the degree of motor connectivity. However, these dependencies have not previously been sufficiently examined. To address this question, we trained Japanese macaque monkeys to vocalize calls or touch a monitor when they encountered visual cues shown on a monitor during an operant conditioning task. Touching the monitor, a hand-and-arm action (i.e. a manual action), requires voluntary motor control, which involves both direct and indirect projections from the motor cortex to motoneurons in macaques [47]. This is equivalent to human vocal/manual control and contrasts with vocal control in non-human primates. We predicted that monkeys would achieve voluntary motor control over vocal production in a different manner from that involved in human speech. A pioneer study by Sutton and colleagues compared motor training between vocal and non-vocal (lever pressing) actions; it revealed a similar ability to learn timing control for both vocal and manual actions [31]. However, the details of the learning processes used in that study were not well documented due to the classical approach for research reports. The current attempt to compare these two motor learning processes facilitates a clearer understanding of the natures of both manual and vocal motor actions; moreover, it enables further characterization of the processes specifically involved in vocal control in macaques.

## 2. Material and methods

### 2.1. Subject information

Four female Japanese macaque monkeys participated in the experiments; two (Pike, Take) were trained for vocal operations, whereas the other two (Toru, Look) were trained for manual operation. All were born in the social group of the Primate Research Institute of Kyoto University, Japan and lived with their mothers before they were moved to individual cages.

### 2.2. Apparatus

The experimental tasks were performed in a custom-made experimental operant box (450 mm W × 450 mm D × 600 mm H) in a sound-attenuated chamber. The monkeys were individually tested in the box. A 22-inch touch-sensitive LCD screen (Dell, Tokyo, Japan; 1024 × 1920-pixel display resolution) was mounted on one side of the experimental box. A universal food dispenser (BUF-310-P100, BIOMEDICA, Osaka, Japan) was placed in the experimental box to provide raisins, a piece of sweet potato or an apple as a food reward. The food dispenser was controlled by computers with USB I/O interfaces (DIO-8/8 (USB) GY, CONTEC, Tokyo, Japan). Stimulus presentation and food dispensing were controlled by a custom-made program.

### 2.3. Training

Two female Japanese macaques (Pike, Take) were trained to execute a vocal action (vocalization); two other female Japanese macaques (Toru, Look) were trained to execute a manual action (touching). Before beginning conditioning in operant chambers with the computer interface, the vocal experiment monkeys were exposed to pre-training stages to increase their motivation for vocal actions to obtain food rewards in their home cages (see pre-training subsection of the electronic supplementary material). Then, the monkeys were trained in operant chambers, which included a mounted touchscreen, microphone and food dispenser; all aspects of the chambers were controlled by a computer outside of the chamber. During training, the monkeys performed tasks in the sound-attenuated chamber, isolated from human influences; importantly, they could not observe the human experimenter. All cues for the task were solely presented though the monitor.

We used a differential reinforcement of low rate (DLR) schedule, which is commonly used for learning tasks during research with monkeys, rats and mice [48,49]. The basic goal of our motor training was differential control of both motor execution and inhibition. The subject was required to execute motor actions as soon as possible after detecting a change from black screen to red screen; additionally, they were required to restrain motor actions during the black screen phase. We continuously monitored the vocal onset and offset of a single call in the vocal condition; similarly, we monitored the timing of a single touch in the manual condition. For the assessment of motor execution, we used the vocalization offset time and touch timing.

At the beginning of each session, a grey screen was shown on the monitor. The first trial of each session began as a screen colour change from grey to red when the monkey looked at the grey screen; this was controlled by the experimenter, who viewed the monkey’s face/gaze direction from a vantage point outside of the sound chamber. In the tasks, monkeys were consistently required to vocalize a single call or touch the screen within 5 s after the presentation of the red screen. If the monkey successfully executed a motor action within 5 s after presentation of the red screen (i.e. we recorded vocal offset timing in the vocal condition or touch actions in the manual condition with a 5-second timeout window), the monitor was immediately changed to a black screen, and the monkey received a food reward with auditory feedback. If the monkey did not execute the vocal/manual action within 5 s, the monitor was changed to a black screen with a buzzer sound as auditory feedback; in this case, the monkey did not receive any food reward. After a fixed period of time in the black screen phase, the next trial was initiated and the red screen was shown. During the fixed time with the black screen, the monkey was required to restrain any vocalization or touching actions. To facilitate differential learning of motor execution and inhibition on the basis of screen cues (red or black), if the monkey failed to restrain motor execution during the restraint phase, the restraint time was prolonged from the time at which vocalization or touching occurred (i.e. the red screen was not shown, and they did not obtain any food reward if they did not restrain vocalizations or touches).

**Figure 1.**
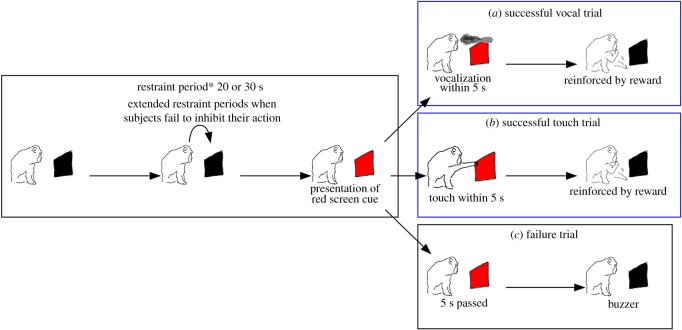
Schematic representation of procedures using differential reinforcement of low rate (DRL). A single trial of the final training stages is shown. The restraint periods were set at 20 or 30 s. During the restraint period, the subject was required to inhibit any action (vocalization for vocal training, touch for touch training), and the restraint times were extended in the case of failure to inhibit action. The subject successfully executed a vocalization (*a*), or a touch (*b*), within 5 s of the red screen cue, and then received a reward. In the case of failure in action execution (*c*), the monitor was blacked out with an auditory feedback buzzer, and the subject did not receive a reward. This trial schedule was cycled during each session.

Manual training was generally easily acquired for both monkeys; however, monkeys in the vocal training group consistently exhibited difficulty in maintaining motivation. This was exemplified by reduced vocal performance during training and failure to generate any sound. To avoid a rapid reduction in motivation, we began training with ‘easy’ parameter settings in the vocal conditions: initially, 5-, 8- or 10-s restraint times were used; when 50% correctness was observed in consecutive sessions, 20- to 30-s restraint times were introduced. For Take, we initially attempted a restraint time of 30 s to match the restraint time for both Pike and Take; however, she could not achieve an improved correct response rate (see electronic supplementary material, figure S1 for restraint time settings). Therefore, we discontinued the use of a 30-s restraint time, and introduced 15-s restraint time to facilitate recovery of motivation; we then extended the restraint time to 20 s to complete the training (electronic supplementary material, figure S1). The number of trials per session was also important for the monkeys to maintain motivation to participate in the vocal tasks; we changed the number of trials depending on each monkey’s motivation (electronic supplementary material, figure S2). Consequently, the final restraint times were set at 30 seconds for Pike and 20 s for Take in a single session, comprising 30 trials (figure 1).

### 2.4. Final criteria for the training completions

Despite the unbalanced progression of restraint times, we aimed to match restraint times during the final training stages for monkeys in both vocal and touch conditions for comparison in the subsequent probe test; restraint times were set at 30 s for Pike and Toru, and 20 s for Take and Look. To assess the completion of training, we used two criteria: correct rate and correct/false index (C/F index). The correct rate comprised a simple calculation of the number of correct trials per total number of trials (i.e. correct trials/total trials); this reflected the degree of motor execution learned by each monkey. The C/F index was defined as follows: C/F index = correct reactions / (correct reactions + false reactions). Note that the number of correct reactions is identical to the number of correct trials used to calculate the correct rate. The C/F index reflects each monkey’s capacity for motor inhibition. Importantly, if the subjects reacted perfectly for all red screen presentations (100% correct rate), that was insufficient to meet both criteria; we also required them to exhibit appropriate reaction inhibition during the restraint phase. When the correct rate reached 90% and the C/F index reached ≥ 0.70 in three consecutive sessions, we regarded the training as complete.

### 2.5. Probe tests

After completing vocal/manual training (meeting both criteria), we introduced probe tests, which comprised trials with five levels of novel restraint times (fixed restraint time multiplied by 0.25, 0.5, 0.75, 1.5 or 2); these were randomly inserted in a session, and the subject was required to generalize the motor action response to the novel restraint times. The purposes of the probe test were to examine the monkeys’ reactions to a novel set of restraint times and to serve as a balance against the difficulty of performance comparison due to the unbalanced histories between vocal and manual training sessions. A single probe test session included 30 trials. The first 10 trials contained restraint times regularly used during training (i.e. 30 s for Pike and Look; 20 s for Take and Toru). Each of the five novel restraint times was then randomly inserted as a probe trial in the latter 20 trials of the session. The probe test was performed once per day; in total, 10 tests were conducted per subject.

### 2.6. Analysis

Direct assessments of the learning processes were seemingly difficult due to the unbalanced histories of the training parameters used for each subject; thus, we examined differences in reaction times between vocal and manual actions during the training session, by using general linear mixed models (GLMMs) with considerations for action type (vocal or manual) as a fixed-effect term and the subject and sessions as random-effect terms with the lmer method in the lme4 package of R. ver 3.4.2. We assessed the estimated parameters of the GLMMs and null model (removing the fixed-effect term from the GLMMs) by using Akaike’s information criterion (AIC) to identify the best model. Next, we performed direct comparisons between probe tests of vocal and manual actions. For the probe tests, we constructed GLMMs with consideration of reaction time as a response variable, restraint times (5 levels: baseline, 0.25 × baseline, 0.5 × baseline, 0.75 × baseline, 1.5 × baseline and 2 × baseline) as fixed-effect terms and session as a random-effect term for each subject. Then, we assessed the statistical significance for estimated parameters of the fixed-effect term by the ANOVA method with the lmerTest package of R. This package enabled us to easily assess the statistical significance of the parameters by using similar ANOVA tests. We subsequently estimated the parameters of the GLMMs to test which probe conditions would differ from baseline conditions when we identified a significant fixed effect in the GLMM-based ANOVA.

## 3. Results

### 3.1. Training progress

**Figure 2.**
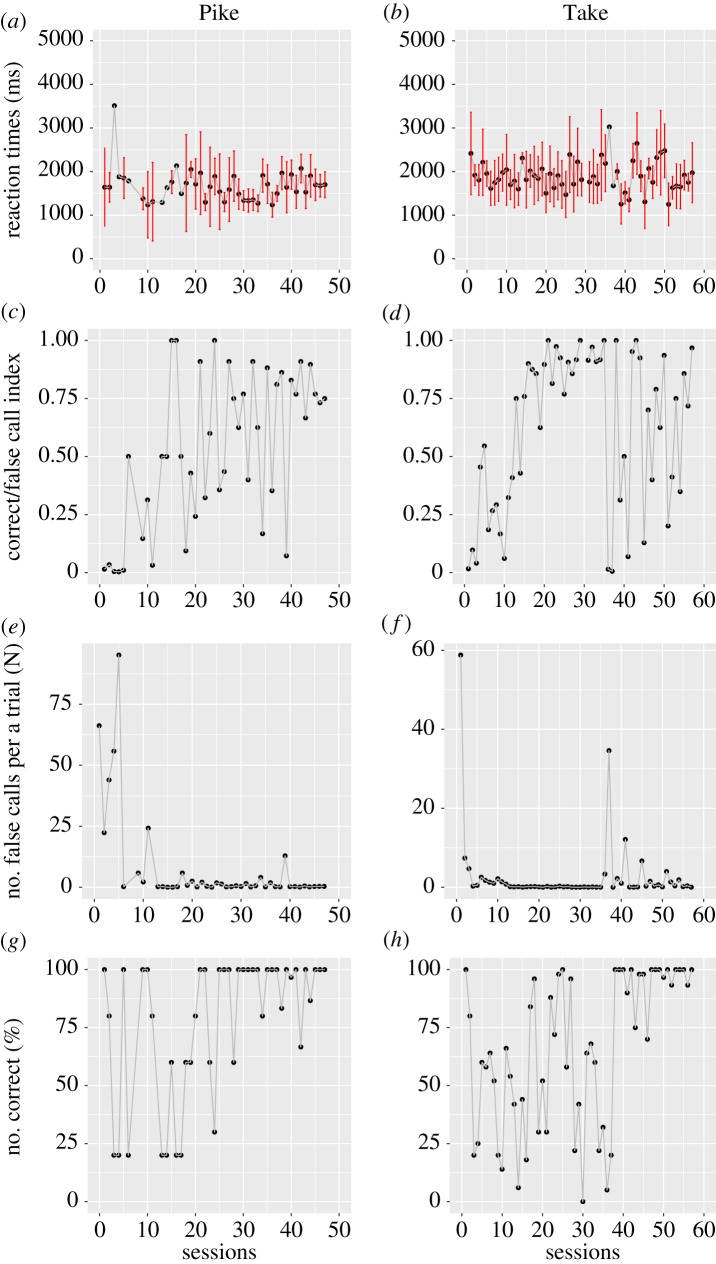
Performance in the progress of vocal training. Learning progress in the vocal tasks after changing from 5 s to 20/30 s of restraint time for Pike and Take. (*a*,*b*) Average reaction times with standard deviations (ms) per session. (*c*,*d*) Correct/false index (C/F index) was defined as follows: C/F index = correct reactions/(correct reactions + false reactions). (*e*,*f*) The number of false calls to be suppressed during the restraint phase. (*g*,*h*) Percentage of successful trials. The learning processes after the change in restraint time (e.g. from 10-sec ITI to 30-sec ITI) in the analysis were shown. Note that restraint time and trial number settings were modified depending on performance and motivation in order to maintain motivation to participate in the vocal task.

**Figure 3.**
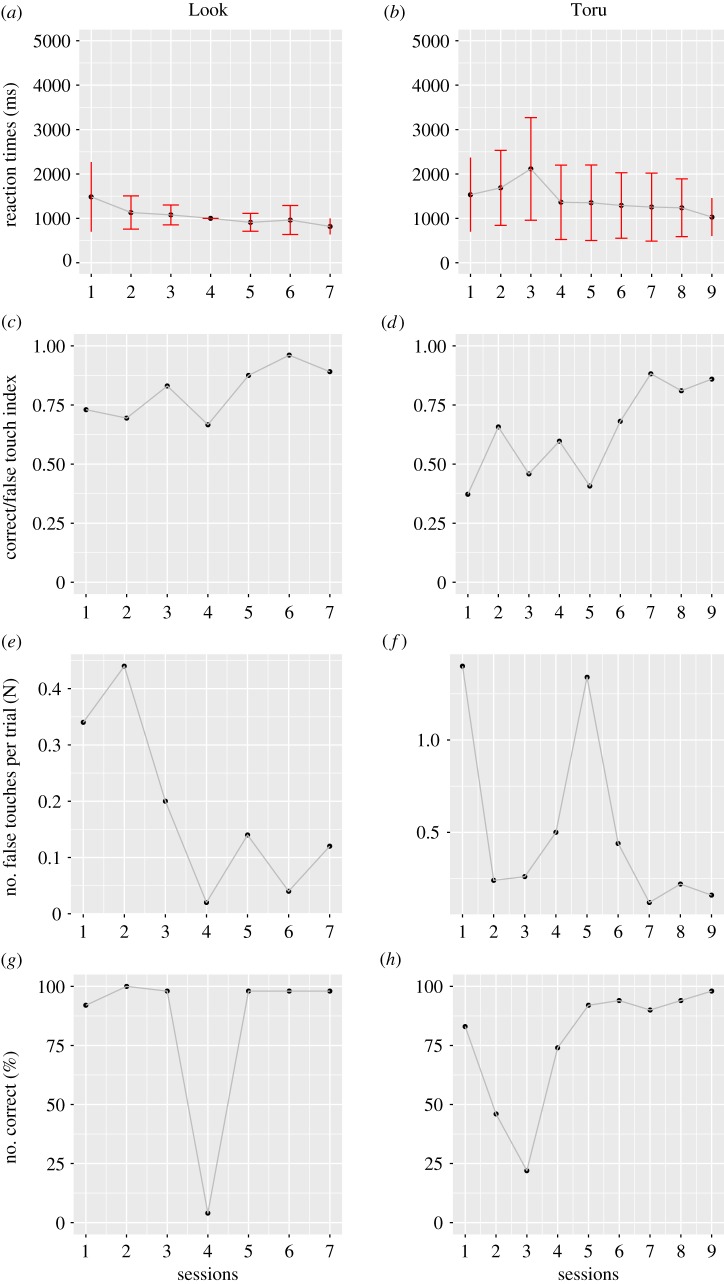
Performance in the progress of touch training. Learning progress in the manual tasks after changing from 5 s to 20/30 s of restraint time for Look and Toru. (*a*,*b*) Average reaction times with standard deviations (ms) per session. (*c*,*d*) Correct/false index (C/F index) were defined as follows: C/F index = correct reactions/(correct reactions + false reactions). (*e*,*f*) The number of false touches to be suppressed during the restraint phase. (*g*,*h*) Percentage of successful trials.

Figures 2 and 3 summarize the learning progress in both vocal and manual tasks, indicating the reaction times of successful trials, the C/F index, numbers of failed actions to be suppressed per trial and the percent of successful trials per session. Failed actions in both vocal and manual tasks increased soon after the initiation of 20–30 s of restraint time (figures 2*e*,*f* , 3*e*,*f*); however, performance immediately improved in the manual task. By contrast, performance improved more slowly in the vocal task: Toru and Look met the criteria by the 9th and 7th sessions, respectively, whereas Take and Pike met the criteria at the 57th and 47th sessions, respectively. Thus, 6–8-fold more sessions were required for training with a novel vocal procedure, compared with a manual task; however, performance was almost identical in the two tasks upon the completion of training. When analysing reaction times in the training process, the best GLMM included the fixed-effect term of action condition (AICs, 45203.39 in GLMM with fixed-effect terms of action condition; 45218.29 in the null model). Furthermore, the GLMM estimated the positive parameter value in vocal tasks (estimated parameter effect of vocal tasks: 528.150 ± 188.135). This suggested that the reaction times were significantly longer in vocal tasks than in manual tasks.

### 3.2. Probe tests

**Figure 4.**
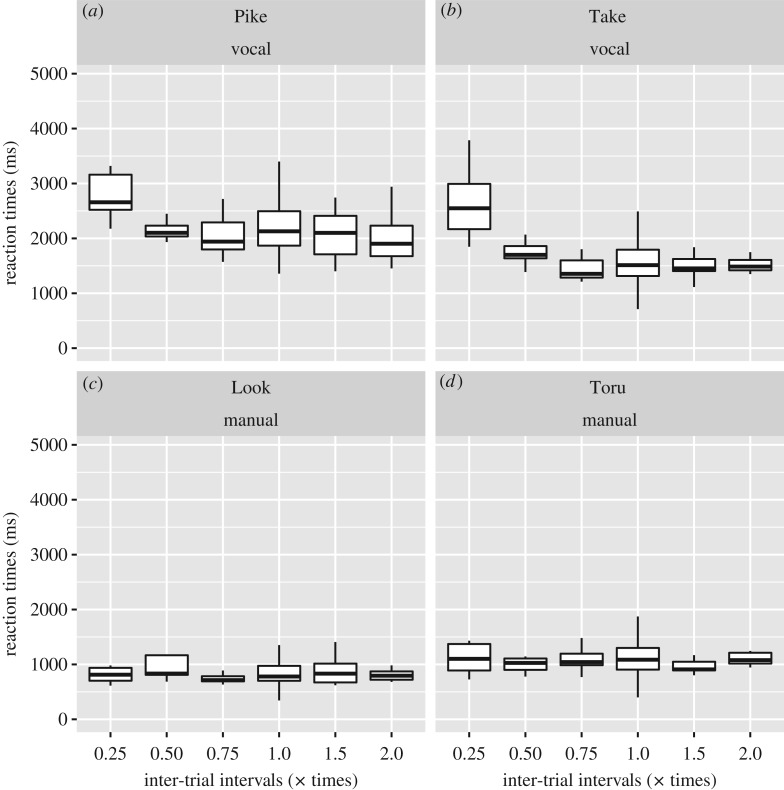
Reaction times of the probe trials and baseline trials, of which inter-trial intervals were set at 20 or 30 s, as used for the training. The *x*-axis shows the inter-trial intervals, where 1 indicates baseline trials, while the other values (0.25, 0.5, 0.75, 1.5 or 2.0) indicate the multiplied conditions of inter-trial intervals for the probe trials. (*a,b*) Show vocal experiments, while the (*c,d*) show touch experiments. Box plots represent the medians (horizontal bold lines), 25th and 75th percentiles (bottom and top of box) and 1.5× interquartile ranges (whiskers).

**Figure 5.**
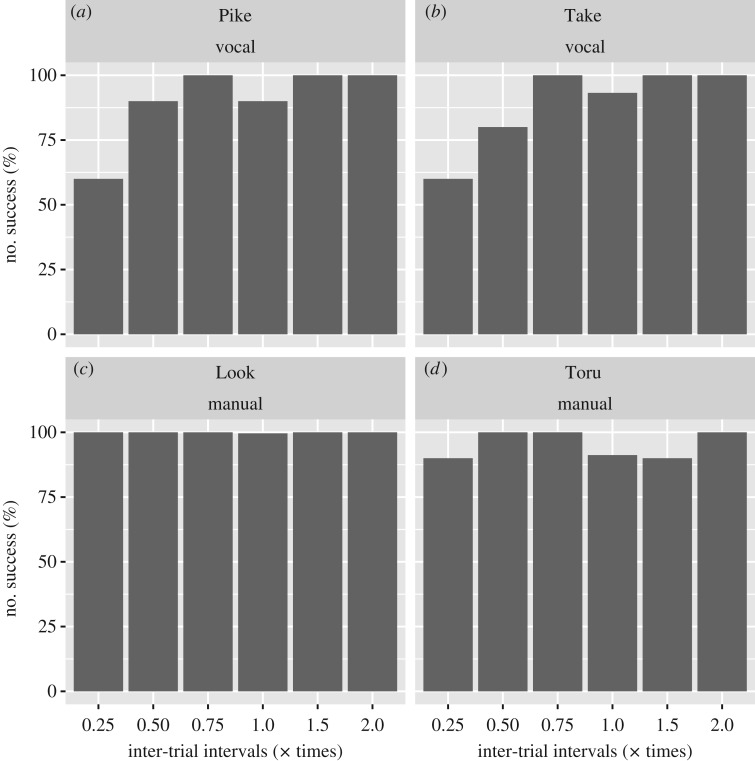
The bar represents the percentage of successful trials for each probe trial condition and baseline trial condition. The *x*-axis represents the inter-trial interval, where 1 indicates baseline trials, while the other values (0.25, 0.5, 0.75, 1.5 or 2.0) indicate multiplied conditions of inter-trial intervals for probe trials. (*a,b*) show vocal experiments, while the (*c,d*) show touch experiments.

Figures 4 and 5 show the results of the probe test. GLMM-based ANOVA by lmerTest revealed no significant effects of novel restraint times for both Toru and Look in the manual task (Toru, *F*_5,70.898_ = 0.72801, *p* = 0.6047; Look, *F*_5,18.205_ = 0.87661, *p* = 0.516), suggesting that there were no significant changes in reaction time between the baseline and each probe trial (figure 4). Look successfully touched within 5 s in all probe trials, and Toru could touch within 5 s for 48 of 50 probe trials.

In contrast, GLMM-based ANOVA for the vocal tasks of Pike and Take revealed a significant effect of the novel restraint times for both Pike and Take (Pike, *F*_5,21.845_ = 3.0953, *p* = 0.02914; Take, *F*_5,24.24_ = 4.924, *p* = 0.003), suggesting that the probe trial condition significantly influenced reaction times. Subsequent analysis of estimated parameters in the full GLMM revealed that reaction time was longer in the probe trial with the restraint time of 0.25 multiplied by the fixed restraint time than at baseline (figure 4; Pike, mean and SD of estimated parameter for reaction time at the × 0.25 condition = 937.04 ± 298.14, *t*_7.58_ = 3.143, *p* = 0.0147; Take, mean and SD of estimated parameters for reaction time at the × 0.25 condition = 1147.69 ± 271.25, *t*_6.06_ = 4.231, *p* = 0.00537, see electronic supplementary material for details of statistical results). Further, the success rate was significantly worse for the probe trial with the restraint time of 0.25 multiplied by the fixed restraint time than for the baseline trial (figure 5; Fisher’s exact test: Pike, odds ratio = 0.169, *p* = 0.0165; Take, odds ratio = 0.111, *p* = 0.00487). Thus, the macaques were not adept at executing a vocalization reaction when the stimuli were suddenly presented at an interval much shorter than that which they had typically encountered.

## 4. Discussion

We could train macaque monkeys to vocalize a single call, as reported in previous operant conditioning studies [28–32,35]. The macaques seemed to ‘homologously’ learn both tasks, largely consistent with the previous comparison study between vocalizations and pressing [31]. However, we found fundamental gaps in the timing control between vocalization and touching. First, the reaction times were different; vocalization required time for motor preparation, but the touch did not. This delay in reaction times was previously observed, such that the vocal reaction was slower than the lever press reaction [31]. In this regard, our data successfully replicated previous evidence. Second, our monkeys required a much longer time to achieve vocal conditioning than touch conditioning. Third, more importantly, they exhibited significant difficulty in executing vocalization in the task with unexpectedly short timing, *despite* extensive motor training. These latter two aspects might differ from the previous classical study [31]. The study by Sutton (1981) concluded that the monkeys’ timing control for vocalization was almost equivalent to that exhibited for manual actions; difficulty in the vocal operant conditioning may not be due to the motor system, but could be related to the experimenter or experimental set-up. Likewise, a review 30 years ago proposed an ‘ecologically appropriate hypothesis,’ which suggested that an understanding of artificial environment behaviour would be predicated upon a knowledge of behaviour in the natural environment. Experiments incorporating appropriate contexts and social reinforcers into the artificial experimental situation might yield the best results [35]. Given this hypothesis, learning difficulties and response delay observed in our demonstrations might be task-dependent problems. A possible factor associated with this inadequacy might be that our subjects were trained without seeing the experimenter, in order to avoid experimenter bias; however, this design devoid of social communication may increase the difficulty encountered by the monkeys in learning the vocal task, whereas it might affect the manual task. Future iterations of these operant conditioning tasks will need to include control of observers’ appearances to better simulate the context of social interaction. Indeed, a recent study showed that vocal learning in marmosets required social context as a key factor in facilitating vocal capabilities [50]; lack of social context might influence motivation. A recent paper regarding operant conditioning reported the importance of age for the success of the training [41]; at younger ages, macaques can more successfully learn a vocal control task. This might reflect subjects’ motivations to participate in an experiment, suggesting that motivation might be a key factor in success.

Another possible cause might be the reinforcement schedule used for our tasks. When compared with schedules in the previous studies, our study used extremely long restraint times, such as 20 or 30 s. Additionally, our DRL schedules required subjects to inhibit their motor actions in specific manners. Importantly, similar studies by Sutton did not include details regarding the inter-trial intervals or the total numbers of actions during sessions, such as the periods before light cues appeared [31]. The delay or learning difficulties in our vocal tasks might be caused by these task-specific parameters in reinforcement schedules; these would likely be related to the biomechanics of vocalization, which has been recently discussed [2,6]. Given the biomechanics of vocal production, animal vocal production requires control of the respiratory system, laryngeal system, tongue, mouth, lips and multiple other body parts in a coordinated fashion. Relatively longer reaction times or difficulty to respond in instances involving shorter timing might be caused by difficulties in coordinating multiple action units. In particular, the respiratory cycle is a fundamental element that constrains the timing of vocalization, because monkeys can only produce calls during expiratory cycles. Respiration is likely to be the primary constraint not under voluntary control. Consequently, vocal timing control is limited by respiratory cycles. By contrast, the manual action is largely independent of the respiratory cycle. This might explain why the monkeys exhibited worse performance in the vocal probe tests when shorter inter-trial intervals were used, while this performance decline was not exhibited in the manual probe tests.

Our training tasks could characterize differences in vocal and manual actions, which previous attempts have not described in detail [28,35,38,40] (i.e. differences in motor preparation and inhibitory control). The uniqueness of our tasks was characterized by the relatively long restraint times in the task schedule. In each task, monkeys were required to discriminate the visual stimulus (monitor colour) and to explicitly organize action control at multiple levels, (i.e. to prepare, inhibit, disinhibit and execute motor actions); importantly, the monkeys exhibit some limitations in vocal motor planning. Difficulties with tasks involving shorter timing suggest the presence of specific deficits, particularly in motor preparation and/or inhibitory control, which are extremely easy to surpass in the motor task in monkeys, as well as in human speech. Thus, systemic acquisition of preparation/inhibitory control with respect to vocal production would be a key event for motor system duality in speech evolution, in terms of motor timing controls.

When considering the comparative brain anatomy of motor systems between monkeys and humans, functional brain expansion of the laryngeal motor cortex might result in system duality between cognitive and emotional control in vocal productions [4,11,51]. Indeed, the control of vocalization timing is one aspect of many motor capabilities underlying human speech. Recent views with regard to vocal production in non-human primates have updated the limited abilities of their speech-related motor abilities. For example, facial expressions such as lip-smacking would be considered a ‘homologous’ motor dimension of speech, as they can be controlled [52–56]. Likewise, vocal tract control has been recently confirmed by great ape vocalizations [46]. In terms of the development of vocal production, marmoset studies have provided innovative evidence that parental influences induce learning with respect to vocal production [50]. Thus, many novel reports have dissected speech ability and have found similar capabilities between non-human primates and humans in a couple of key vocal features [6]. However, cortico-motor connections represent a critical source of difference and may serve as a plausible candidate for the development of novel motor functions in all animals. Motor deficits in vocal timing control might be reflected by their neural circuits, which is consistent with recent unit-recording studies [4,5,40,42]. The concept that expansions of direct cortico-motor neuronal connections might provide new voluntary actions would explain other evolutions of the motor system in hominids, non-human primates and non-primate mammals [47]. Specifically, when considering the dexterity of forelimb, hand or finger voluntary actions in mammals, the extent of dexterity is well-correlated with expansion of direct cortico-motoneuronal connections, an essential system for dexterity in manual actions, in motor areas that map to forelimb, hands and finger controls [47,57–60]. Likewise, humans might have acquired voluntary vocal control due to an expansion of direct cortico-motoneuronal connections in the human laryngeal motor cortex (LMC) located in Broadman Area (BA) 4 and 6 in humans, which are absent in monkey LMC (solely exhibiting BA6 of the premotor cortex) [58,60]; this suggests that emergence of cortico-motoneuronal connections in the LMC might have evolutionarily occurred after the emergence of a now-extinct non-human primate–hominid ancestor [10,11]. The parallel phenomena of motor cortex expansions provide novel functions of top-down cognitive control for motor execution in primate evolution; the motor innovations might first occur in forelimb control between non-primates and primates, and next in vocal control between non-human primates and humans [10,11]. As a possible evolutionary scenario, motor innovation might first have occurred in forelimb control during the evolution of primates from non-primates. A similar evolution in vocal control may have then occurred in the evolution of humans from non-human primates. The LMC connection is one of the possible neuroanatomical changes that could have led to the emergence of speech, but is not enough for speech. The fine motor control of jaws, lips, tongue and diaphragm should also evolve. Together with the motor control systems of other elements, such as facial muscles, vocal tracts and respiration, the speech might have then emerged as a unique form of motor systems integration.

## Supplementary Material

supporting methods and results

## Data accessibility

All data are available in online Supplementary Materials or Dryad: https://datadryad.org/resource/doi:10.5061/dryad.6v7j674 [61].
