# Supplementary material for: From hand to mouth: monkeys require greater effort in motor preparation for voluntary control of vocalization than for manual actions
Source: R Soc Open Sci. 2018 Nov 28;5(11):180879. doi: 10.1098/rsos.180879 (PMC6281949; doi:10.1098/rsos.180879)
Supplement: supporting methods and results [file rsos180879supp1.pdf]

Supplementary Informaion of ”From hand to mouth: Monkeys require greater effort in motor preparation for voluntary control of vocalisation than for manual actions”

Hiroki Koda[1,*], Takumi Kunieda[1], and Takeshi Nishimura[1]

[1]Primate Research Institute, Kyoto University
*koda.hiroki.7a@kyoto-u.ac.jp

1 Supplementary Materials and Methods

1.1 Pre-training for vocal conditioning.

Spontaneous vocalizations are lower than are those for manual actions like touches in Japanese macaques generally, particularly in isolated environments. This limits their opportunities for associative learning of cue detection and vocal motor reactions. For vocal training, monkeys should be prepared to increase the rate of spontaneous vocal production. Therefore, we set three pre-stages for training monkeys to acquire associative learning between cue detection and motor execution, following our previous procedure for vocal operant conditioning in a small ape [1]. First, we always reinforced their vocalizations by giving a food reward without any associative cues in the cage room where they live. Standing in front of the monkey cage, the trainer waited for the subjects vocalizations, simultaneously showing food in the trainers hand. Once the monkey vocalized, the trainer provided a food reward immediately with sound feedback from a clicker, and repeated these attempts daily for at least 30 minutes once a day. In the next stage, their vocalizations were reinforced when the trainer showed an A4-size red card together with food in the hand. This was done to train a three-term contingency, i.e., an association among cue presentation, vocalization, and reward. These pre-stages for reward learning are necessary to sufficiently increase the motivation to vocalize. Then, we showed only the red card to induce vocalizations isolated from their arousal modulated

by directly seeing a food reward. Consequently, the subjects increased their rates of spontaneous vocalization, and learned to vocalize when they saw the red card. After these pre-stages, we trained them in the sound chamber. Three and two months were taken for Pike and Take to pass the pre-stages, respectively.

1.2 Training parameter settings

To avoid rapid reduction in motivation, we began training with easy parameter settings in the vocal conditions: initially, 5-, 8-, or 10-second restraint times were used; when 50% correctness was observed in consecutive sessions, 20- to 30-second restraint times were introduced. For Take, we initially attempted a restraint time of 30 seconds to match the restraint time for both Pike and Take; however, she could not achieve an improved correct response rate (see Figure S1 for restraint time settings). Therefore, we discontinued the use of a 30-second restraint time, and introduced 15-second restraint time to facilitate recovery of motivation; we then extended the restraint time to 20 seconds to complete the training (Figure S1). The number of trials per session was also important for the monkeys to maintain motivation to participate in the vocal tasks; we changed the number of trials depending on each monkeys motivation (Figure S2). Consequently, the final restraint times were set at 30 seconds for Pike and 20 seconds for Take in a single session, comprising 30 trials.

References

[1] Koda H, Oyakawa C, Kato A, Masataka N. 2007 Experimental evidence for the volitional control of vocal production in an immature gibbon. *Behaviour* **144**, 681–692.

2 Supplementary Results

2.1 Statistical results of GLMM-based ANOVAs

estimated parameters	mean	sd	*t*	*df*	*p*
intercept(baseline restraint times)	2245.139	87.072	9.006	25.785	9.48e-10
×0.25	937.04	298.14	3.143	7.58	0.0147
×0.50	-66.056	176.950	20.632	-0.373	0.7127
×0.75	-213.739	147.106	189.492	-1.453	0.1479
×1.50	-176.939	149.314	83.625	-1.185	0.2394
×2.00	-223.639	155.696	19.982	-1.436	0.1664

Supplementary Table S 1: Pike's parameter estimations by GLMM by lmerTest methods of R. In the model, reaction times of the baseline restraint time trials were set as intercept, and the difference between each of restraint time condition and baselines were estimated.

estimated parameters	mean	sd	*t*	*df*	*p*
intercept(baseline restraint times)	1594.374	57.600	9.254	27.680	3.24e-10
×0.25	1147.69	271.25	4.231	6.06	0.00537
×0.50	256.222	214.076	13.809	1.197	0.25150
×0.75	-161.374	152.452	126.342	-1.059	0.29184
×1.50	-117.474	151.771	160.727	-0.774	0.44005
×2.00	-34.674	151.479	194.636	-0.229	0.81918

Supplementary Table S 2: Take's parameter estimations by GLMM by lmerTest methods of R. In the model, reaction times of the baseline restraint time trials were set as intercept, and the difference between each of restraint time condition and baselines were estimated.

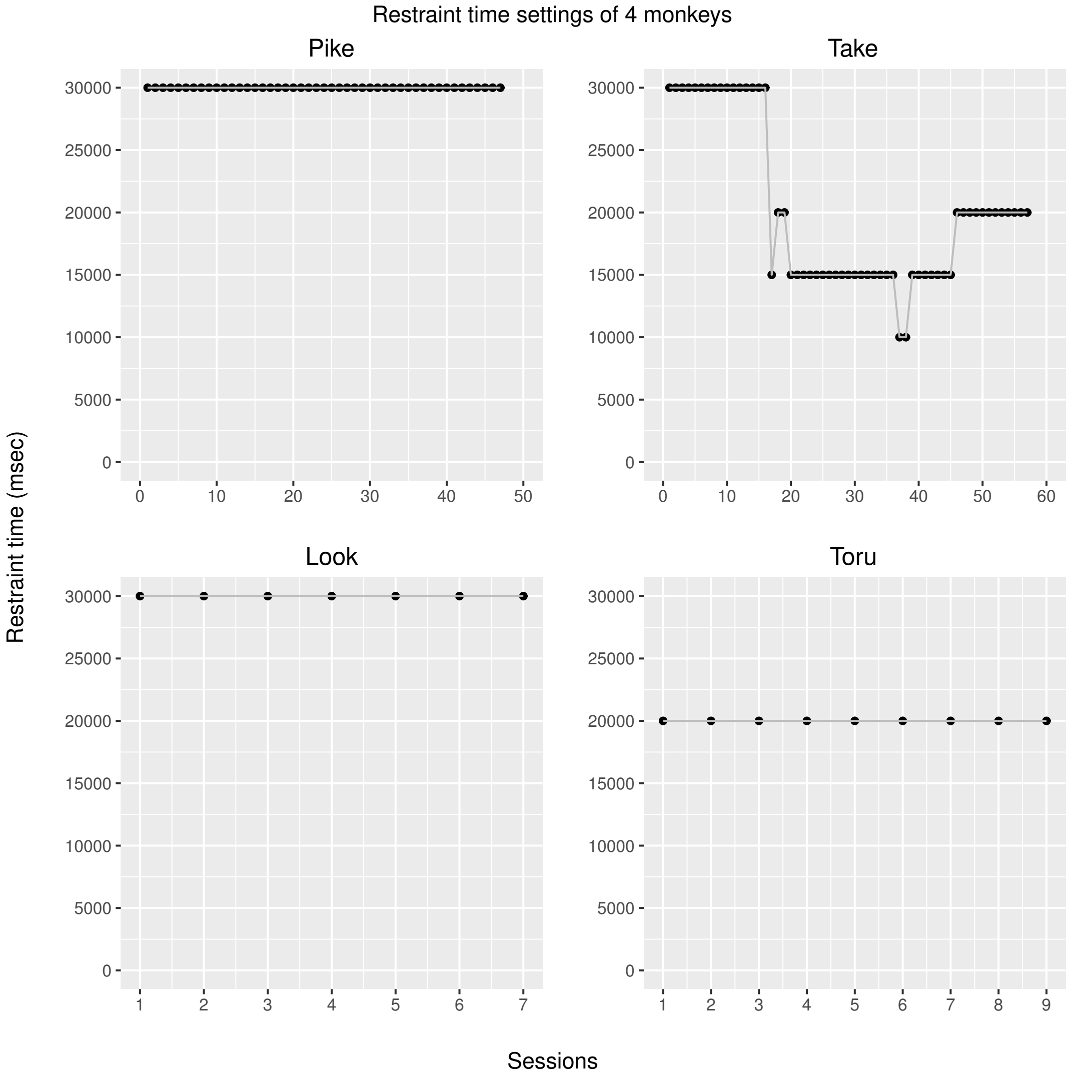

Figure S1: Restraint time settings were shown with the training progress after changing to 20/30-seconds restraint times, for vocal subjects, Pike and Take (top) and manual subjects, Look and Toru (bottom). Pike and Take were first trained 30-seconds, and Pike gradually learned the vocal task; however, Take could not improve her correct response rate (see Figure 2h), and therefore, we reduced from 30 to 15 seconds for training, and then we extended again from 15 to 20 seconds to complete her training. By contrast, manual task was easier to be learned. To match the restraint time of these vocal subjects with that of the touch subjects, the restraint time was continuously set at 30 seconds for Toru, and 20 seconds for Look.

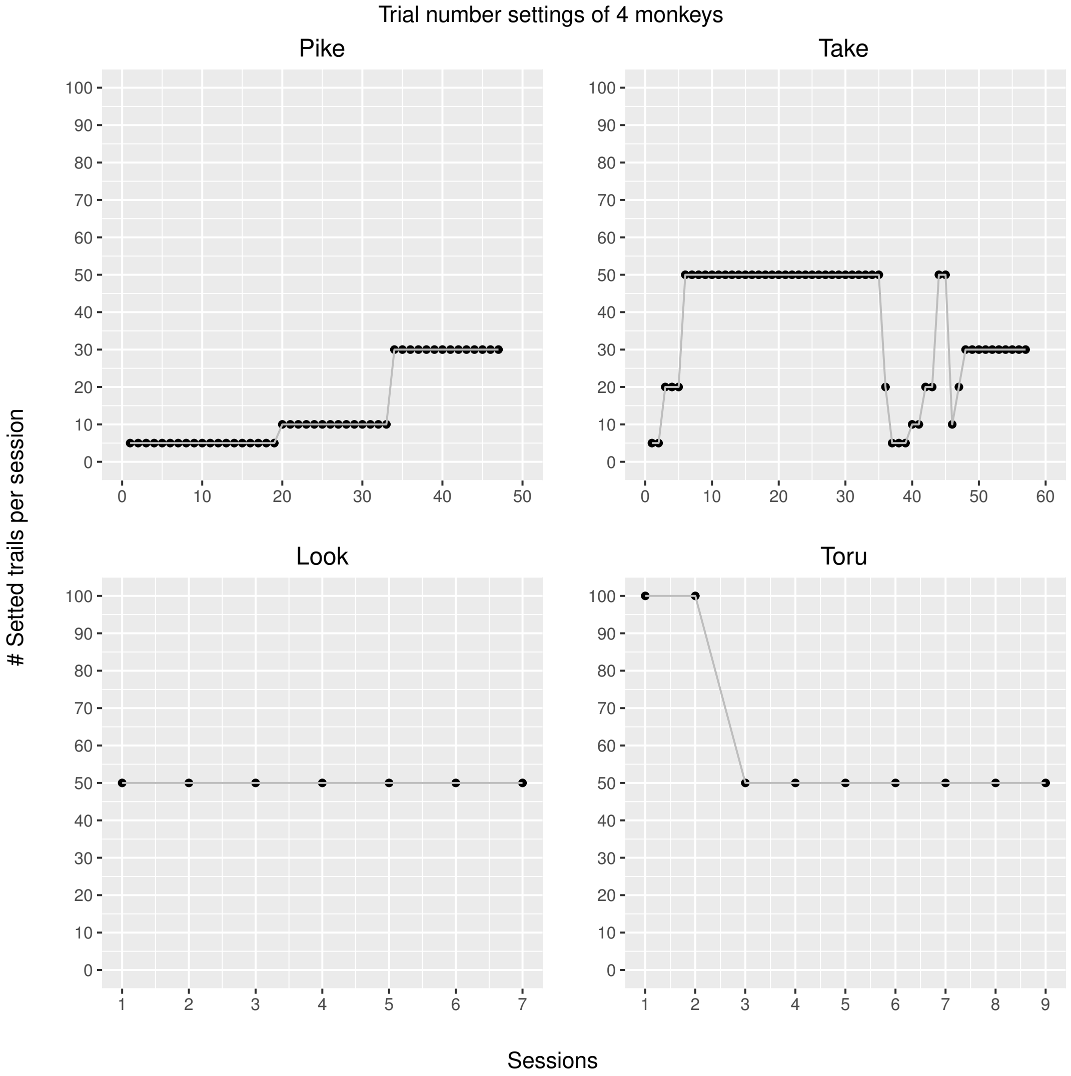

Figure S2: Trial number settings were shown with the training progress after changing to 20/30-seconds restraint times, for vocal subjects, Pike and Take (top) and manual subjects, Look and Toru (bottom). The trial numbers per session were important to maintain their motivation to participate in vocal tasks, we changed the trial numbers depending on the subject's motivation. Particularly, for vocal tasks, we carefully set the trial number to avoid the extinction of the vocalizations due to the crucial difficulty of vocal tasks. We started from 5 trials, and increased/decreased the trial numbers depending on the subject motivations.
